# Editorial: Peptidyl-prolyl *cis/trans* isomerases (PPIases) in host-pathogen interactions

**DOI:** 10.3389/fcimb.2022.1097771

**Published:** 2022-12-07

**Authors:** Michael Steinert

**Affiliations:** Institut für Mikrobiologie, Technische Universität Braunschweig, Braunschweig, Germany

**Keywords:** peptidyl-prolyl *cis/trans* isomerases, FKBP, cyclophilin, parvulin, host-pathogen interaction, therapeutics

This Research Topic on peptidyl-proly *cis/trans* isomerases (PPIases) is dedicated to Gunter Fischer who discovered these ubiquitous enzymes in the year 1984 (Fischer et al., 1984). His pioneering work and several decades of very fruitful PPIase research in prokaryotes and eukaryotes inspired many scientific disciplines and opened up the possibility of treating a wide range of diseases (Kolos et al., 2018; Pomplun et al., 2018; Scheuplein et al., 2020; Iwasaki et al., 2022). PPIases catalytically accelerate the interconversion of the energetically favored *cis* and *trans* isomers of prolyl bonds and interact with specific substrate proteins (Schiene-Fischer et al., 2011). The superfamily of PPIases comprise FK506-binding proteins (FKBPs), cyclophilins, and parvulins and controls a multitude of processes involved in host-pathogen interactions (Ünal and Steinert, 2014). Eukaryotic PPIases play key roles in cancer, neurodegeneration, psychiatric disorders, bacterial and viral infections (Lee et al., 2011; Ünal and Steinert, 2014; Blair et al., 2015), while bacterial PPIases are involved in metabolism, virulence, and multiple stress responses (Rasch et al., 2018; Ünal et al., 2019).

Although cyclophilins, FKBPs and parvulins are known to influence physiological and pathophysiological processes in pathogens, hosts, and their interactions, only a few natural substrates of these PPIases have been identified (Ünal et al., 2019). However, new methods in interactome research and machine learning seem to improve this situation (Karagöz et al., 2022).

This Research Topic provides representative examples for viral, toxin, bacterial, and parasitic host-pathogen interactions. The compiled articles shed light on how PPIases control fundamental properties of their target proteins such as bioactivity, localization or stability, but also evaluate innovative therapeutic strategies which address host and/or pathogen PPIases.

In the Brief Research Report “*Effects of immunophilin inhibitors and non-immunosuppressive analogs on coronavirus replication in human infection models*” Berthold et al. demonstrates that the immunophilin inhibitor cyclosporine A (CsA) inhibits coronavirus replication at non-toxic low-micromolar concentrations. This effect was independent of the immunosuppressive effect of CsA, since the non-immunosuppressive analog Alisporivir (ALV) was similarly effective in various cellular infection models. The immunosuppressive FK506 exhibited inconsistent cell-type specific inhibitory effects on corona replication, while FK506-derived non-immunosuppressive analogs revealed no inhibitory effects, respectively. The lack of efficacy for the non-immunosuppressive FK506 analogs suggests a role of the immunosuppressive calcineurin binding domain, which is substituted.

In humans, eighteen cyclophilin and sixteen FKBP isoenzymes and numerous parvulins including Pin1 have been described and the importance of cyclophilin CypA during HIV and HCV infections is well established (Watashi et al., 2008; Lim et al., 2011). Nevertheless there is a lack of information to what extent and how human PPIases exactly facilitate bacterial toxin activity. In “*Requirement of peptidyl-prolyl cis/trans isomerases and chaperones for cellular uptake of bacterial AB-type toxins*” Ernst reports about the role of PPIases of the cyclophilin and FK506 binding protein families during uptake of *Clostridium botulinum* C2 toxin, *Clostridium perfringens* iota toxin, *Clostridioides difficile* CDT toxin, diphtheria toxin, pertussis toxin and cholera toxin. Like viruses bacterial AB-type toxins act independently as a particle. In this review the hypothesis that host cell PPIases and heat shock proteins are required to facilitate the directed translocation of the enzyme subunits to the cytosol and to assist their refolding into an active conformation is critically evaluated. To what extent bacterial PPIases of Gram-negative and -positive bacteria additionally modulate these processes and if intoxicated patients would benefit from PPIase inhibitors have to be revealed in future research (Ünal et al., 2018; Ünal et al., 2019).

In the Mini Review entitled “*Update on the Neisseria macrophage infectivity potentiator-like PPIase protein*” Christodoulides highlights the potential of Mip as drug target and vaccine candidate for gonorrhoea and meningitidis infections. The analysis of recent results suggests that Mip should be explored for the first generation of modern antigen-specific gonococcal vaccines and structural vaccinology approaches.

Since PPIases are also relevant in eukaryotes and parasites often have multiple PPIases “*Structural insights into Plasmodium PPIases*”, as reviewed by Rajan and Yoon, may pave the way for new drugs. The authors portray the structures of *Plasmodium* PPIases, their inhibitor complexes and come to the conclusion that identifying the natural substrates and characterizing the binding proteins would help to design more specific inhibitors and non-immunsuppressive drugs.

In conclusion, this Research Topic highlights the relevance of bacterial and parasitic PPIases in virulence and underlines that host PPIases are also implicated in viral, bacterial, and parasitic infections. Moreover, it becomes increasingly clear that host and bacterial PPIases are promising drug targets to fight infections.

## Author contributions

The author confirms being the sole contributor of this work and has approved it for publication.
